# Supplementary figures and images for: Genotypic diversity and plasticity of root system architecture to nitrogen availability in oilseed rape
Source: PLoS One. 2021 May 20;16(5):e0250966. doi: 10.1371/journal.pone.0250966 (PMC8136655; doi:10.1371/journal.pone.0250966)

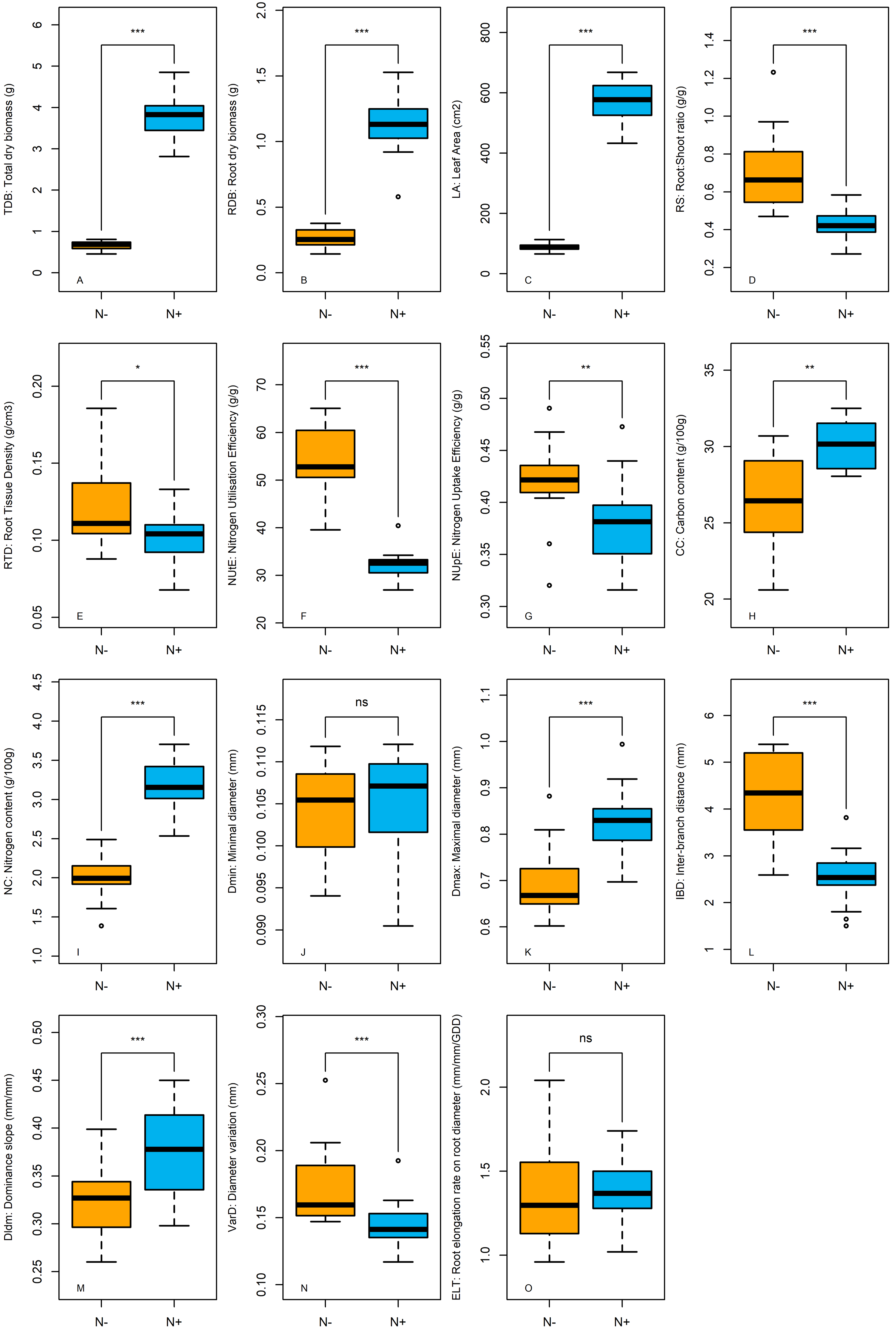

Supplement: S1 Fig — Size and color of the squares indicate the value of the Pearson’s correlation coefficient, at significant levels ≤ 0.05. Non-significant correlations are represented by empty cells. PA and RSA traits are shown in black and red, respectively. (TIF) [file pone.0250966.s001.tif]

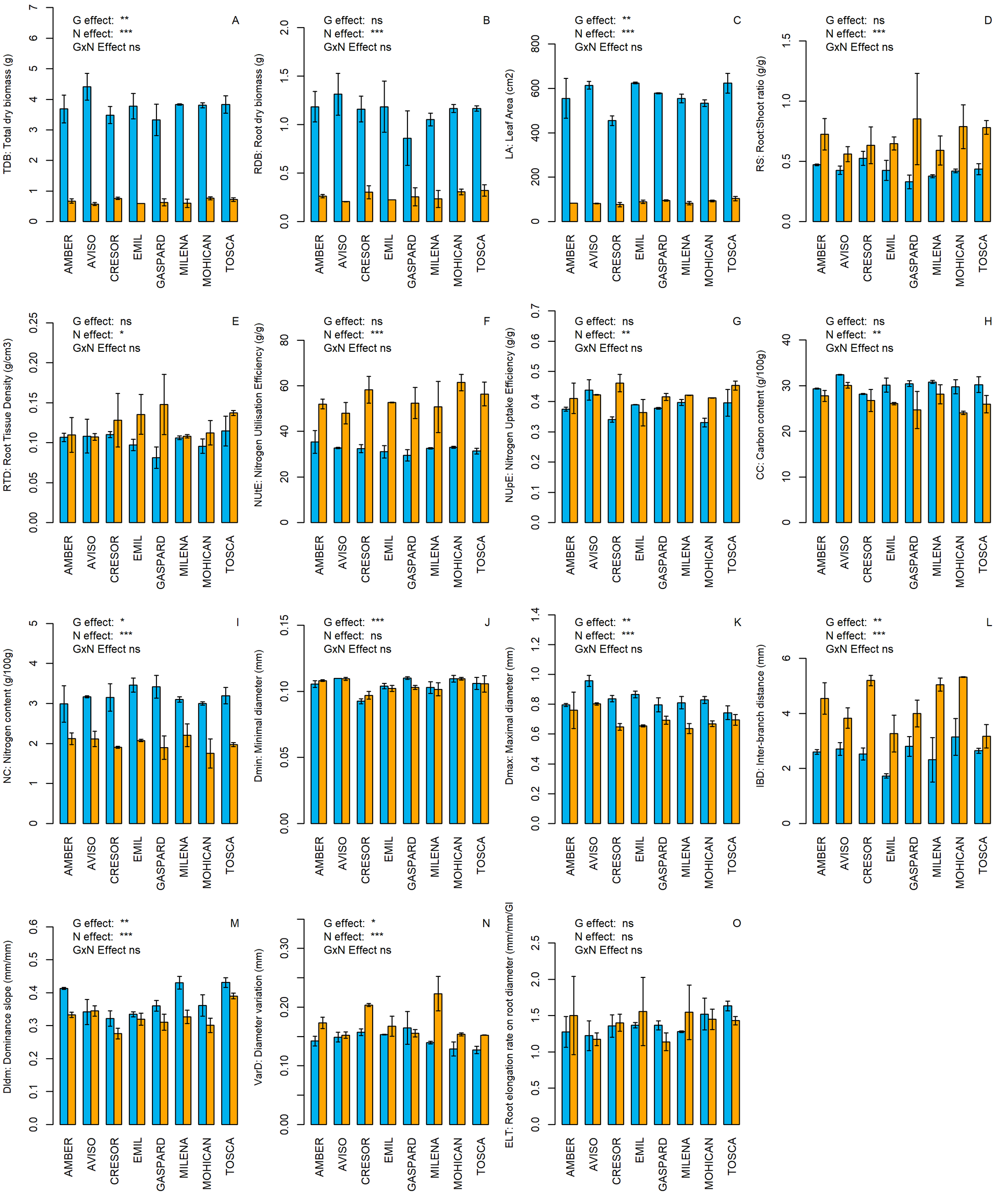

Supplement: S2 Fig — Total dry biomass (A), Root dry biomass (B), Leaf area (C), Root:Shoot ratio (D), Root tissue density (E), Nitrogen Utilization Efficiency (F), Nitrogen Uptake Efficiency (G), Carbon content (H), Nitrogen content (I), Root minimal diameter (J), root maximal diameter (K), Inter-Branch distance (L), Root system dominance (M) Lateral root diameter variation (N) and Root elongation rate per root diameter (O). Significant differences between the two N nutritions were listed on the top of each graph and were determined through an ANCOVA (***: pvalue < 0.001; **: pvalue < 0.01; *: pvalue < 0.05; ns: pvalue > 0.05). Orange boxes stand for N- plants and blue boxes stand for N+ plants (n = 16). (TIF) [file pone.0250966.s002.tif]

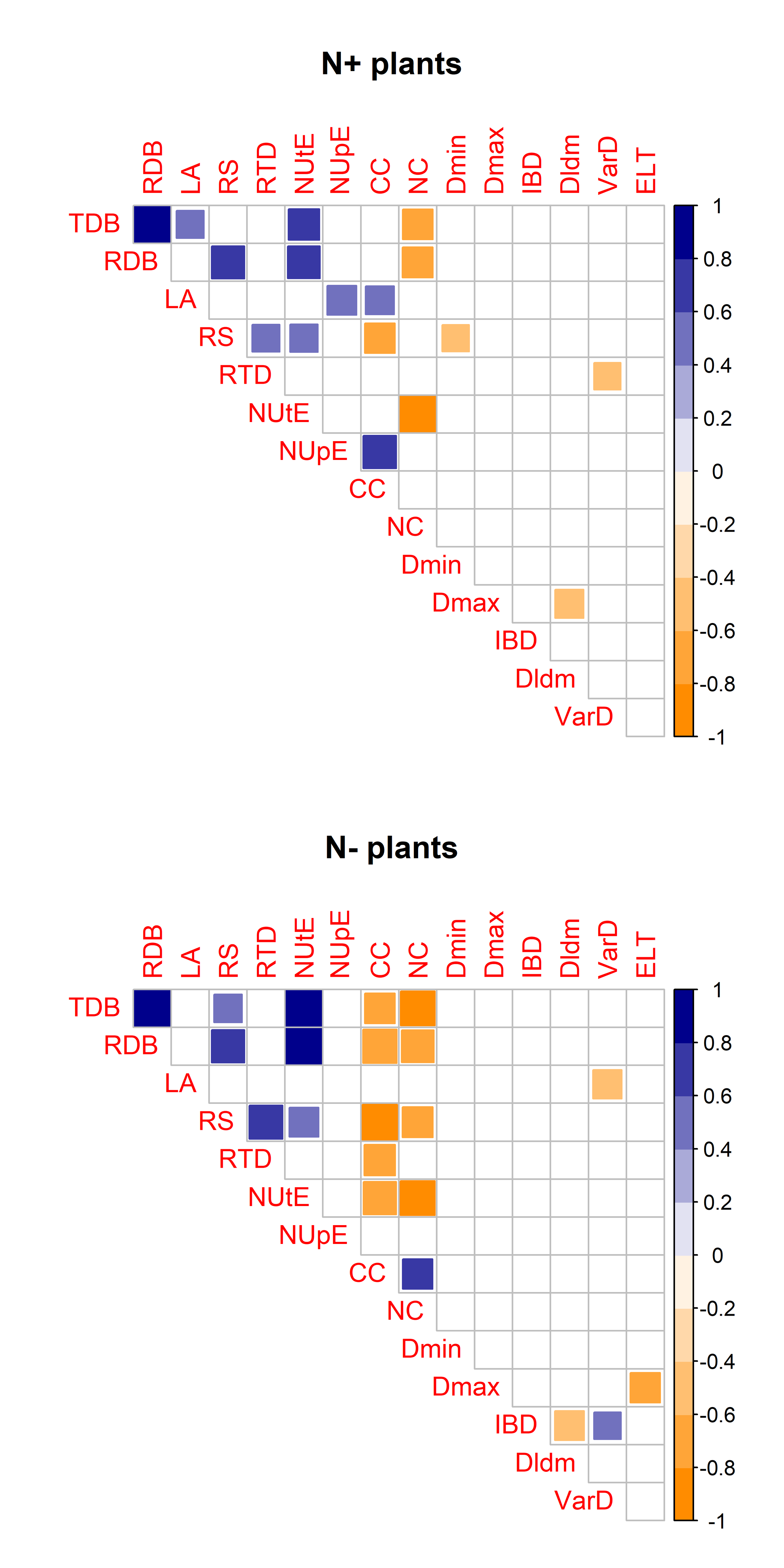

Supplement: S3 Fig — Barplots showing mean trait values for each genotype under the two N nutritions. Total dry biomass (A), Root dry biomass (B), Leaf area (C), Root:Shoot ratio (D), Root tissue density (E), Nitrogen Utilization Efficiency (F), Nitrogen Uptake Efficiency (G), Carbon content (H), Nitrogen content (I), Root minimal diameter (J), root maximal diameter (K), Inter-Branch distance (L), Root system dominance (M) Lateral root diameter variation (N) and Root elongation rate per root diameter (O). Significant effect of genotype, N treatment and interaction between genotype and N treatment were assessed through an ANCOVA (***: pvalue < 0.001; **: pvalue < 0.01; *: pvalue < 0.05; ns: pvalue > 0.05). Orange bars stand for N- plants and blue bars stand for N+ plants (n = 2). Error bars represent standard errors (+/- SE). (TIF) [file pone.0250966.s003.tif]
