# Supplementary material for: Genotypic diversity and plasticity of root system architecture to nitrogen availability in oilseed rape
Source: PLoS One. 2021 May 20;16(5):e0250966. doi: 10.1371/journal.pone.0250966 (PMC8136655; doi:10.1371/journal.pone.0250966)
Supplement: S1 Table — (DOCX) [file pone.0250966.s004.docx]

**S1 Table. Composition of each of the two nutritive solutions used in the experimentation.**

| **Component** | **Concentration (mM)** | |
| --- | --- | --- |
|  | N+ solution | N- solution |
| KCl | 3 | 3 |
| KH_2_PO_4_ | 0,498 | 0,498 |
| MgSO_4_, 7H_2_O | 1 | 1 |
| CaCl_2_ | 2,5 | 2,5 |
| MnCl_2_, H_2_O | 0,010058 | 0,010058 |
| ZnSO_4_, 5H_2_O | 0,0010433 | 0,0010433 |
| CuSO_4_, 5H_2_O | 0,0004806 | 0,0004806 |
| H_3_BO_3_ | 0,0307294 | 0,0307294 |
| (NH_4_)_6_MO_7_O_24_, 4H_2_O | 9,71E-05 | 9,71E-05 |
| CoSO_4_, 6H_2_O | 0,0005 | 0,0005 |
| Fe EDTA Na | 0,0270144 | 0,0270144 |
| K(NO_3_) | 5 | 0,5 |
| Ca(NO_3_)_2_, 4H_2_0 | 2,5 | 0,25 |
