# Supplementary material for: Genotypic diversity and plasticity of root system architecture to nitrogen availability in oilseed rape
Source: PLoS One. 2021 May 20;16(5):e0250966. doi: 10.1371/journal.pone.0250966 (PMC8136655; doi:10.1371/journal.pone.0250966)
Supplement: S2 Table — Mean, standard deviation (sd) and coefficient of variation (cv) were calculated per N treatment (n = 16). (DOCX) [file pone.0250966.s005.docx]

S2 Table: Descriptive statistics of all traits. Mean, standard deviation (sd) and coefficient of variation (cv) were calculated per N treatment (n=16).

|  | **N- treatment** | | | **N+ treatment** | | |
| --- | --- | --- | --- | --- | --- | --- |
| **Statistic** | *mean* | *sd* | *cv* | *mean* | *sd* | *cv* |
| **TDB** | 0.66 | 0.11 | 0.16 | 3.77 | 0.48 | 0.13 |
| **RDB** | 0.26 | 0.07 | 0.27 | 1.13 | 0.22 | 0.19 |
| **LA** | 87.96 | 10.81 | 0.12 | 566.97 | 67.62 | 0.12 |
| **RS** | 0.7 | 0.21 | 0.3 | 0.43 | 0.07 | 0.17 |
| **RTD** | 0.12 | 0.03 | 0.22 | 0.1 | 0.02 | 0.16 |
| **NUtE** | 54.01 | 7.36 | 0.14 | 32.26 | 2.95 | 0.09 |
| **NUpE** | 0.42 | 0.04 | 0.09 | 0.38 | 0.04 | 0.1 |
| **CC** | 26.68 | 2.81 | 0.11 | 30.17 | 1.58 | 0.05 |
| **NC** | 2 | 0.26 | 0.13 | 3.18 | 0.3 | 0.1 |
| **Dmin** | 0.1 | 0.01 | 0.05 | 0.1 | 0.01 | 0.06 |
| **Dmax** | 0.69 | 0.08 | 0.11 | 0.83 | 0.07 | 0.08 |
| **IBD** | 4.3 | 0.93 | 0.22 | 2.56 | 0.58 | 0.23 |
| **Dldm** | 0.33 | 0.04 | 0.11 | 0.37 | 0.05 | 0.13 |
| **VarD** | 0.17 | 0.03 | 0.17 | 0.15 | 0.02 | 0.12 |
| **ELT** | 1.4 | 0.35 | 0.25 | 1.38 | 0.2 | 0.14 |
