# Supplementary material for: Genotypic diversity and plasticity of root system architecture to nitrogen availability in oilseed rape
Source: PLoS One. 2021 May 20;16(5):e0250966. doi: 10.1371/journal.pone.0250966 (PMC8136655; doi:10.1371/journal.pone.0250966)
Supplement: S3 Table — Mean, standard deviation (sd) and variation coefficient (cv) were calculated per genotype for each trait (n = 2). (DOCX) [file pone.0250966.s006.docx]

S3 Table: Descriptive statistics of N+ plants. Mean, standard deviation (sd) and variation coefficient (cv) were calculated per genotype for each trait (n=2).

| Genotype | **AMBER** | | | **AVISO** | | | **CRESOR** | | | **EMIL** | | | **GASPARD** | | | **MILENA** | | | **MOHICAN** | | | **TOSCA** | | |
| --- | --- | --- | --- | --- | --- | --- | --- | --- | --- | --- | --- | --- | --- | --- | --- | --- | --- | --- | --- | --- | --- | --- | --- | --- |
| **Trait** | *mean* | *sd* | *cv* | *mean* | *sd* | *cv* | *mean* | *sd* | *cv* | *mean* | *sd* | *cv* | *mean* | *sd* | *cv* | *mean* | *sd* | *cv* | *mean* | *sd* | *cv* | *mean* | *sd* | *cv* |
| **TDB** | 3.68 | 0.64 | 0.17 | 4.41 | 0.62 | 0.14 | 3.48 | 0.39 | 0.11 | 3.77 | 0.59 | 0.16 | 3.33 | 0.73 | 0.22 | 3.83 | 0.03 | 0.01 | 3.8 | 0.12 | 0.03 | 3.83 | 0.41 | 0.11 |
| **RDB** | 1.18 | 0.22 | 0.19 | 1.31 | 0.31 | 0.23 | 1.16 | 0.19 | 0.16 | 1.18 | 0.37 | 0.31 | 0.86 | 0.4 | 0.46 | 1.05 | 0.09 | 0.09 | 1.17 | 0.06 | 0.05 | 1.17 | 0.04 | 0.03 |
| **LA** | 555 | 126 | 0.23 | 613 | 24.4 | 0.04 | 454 | 30.3 | 0.07 | 624 | 5.42 | 0.01 | 578 | 2.73 | 0 | 554 | 27.6 | 0.05 | 532 | 21.4 | 0.04 | 623 | 63.4 | 0.1 |
| **RS** | 0.47 | 0.01 | 0.02 | 0.43 | 0.05 | 0.11 | 0.53 | 0.08 | 0.16 | 0.43 | 0.12 | 0.28 | 0.33 | 0.08 | 0.25 | 0.38 | 0.02 | 0.04 | 0.42 | 0.02 | 0.05 | 0.44 | 0.06 | 0.15 |
| **RTD** | 0.11 | 0.01 | 0.07 | 0.11 | 0.03 | 0.28 | 0.11 | 0.01 | 0.05 | 0.1 | 0.01 | 0.11 | 0.08 | 0.02 | 0.23 | 0.11 | 0 | 0.03 | 0.1 | 0.01 | 0.13 | 0.11 | 0.03 | 0.23 |
| **NUtE** | 35.37 | 7.11 | 0.2 | 32.76 | 0.43 | 0.01 | 32.46 | 2.48 | 0.08 | 31.05 | 3.92 | 0.13 | 29.49 | 3.61 | 0.12 | 32.54 | 0.38 | 0.01 | 33.05 | 0.62 | 0.02 | 31.36 | 1.82 | 0.06 |
| **NUpE** | 0.38 | 0.01 | 0.03 | 0.44 | 0.05 | 0.11 | 0.34 | 0.01 | 0.04 | 0.39 | 0 | 0 | 0.38 | 0 | 0.01 | 0.4 | 0.01 | 0.04 | 0.33 | 0.02 | 0.06 | 0.4 | 0.06 | 0.16 |
| **CC** | 29.35 | 0.13 | 0 | 32.43 | 0.09 | 0 | 28.16 | 0.16 | 0.01 | 30.15 | 2.21 | 0.07 | 30.45 | 0.91 | 0.03 | 30.82 | 0.44 | 0.01 | 29.79 | 2.19 | 0.07 | 30.23 | 2.42 | 0.08 |
| **NC** | 2.99 | 0.64 | 0.21 | 3.17 | 0.03 | 0.01 | 3.15 | 0.49 | 0.15 | 3.46 | 0.25 | 0.07 | 3.42 | 0.4 | 0.12 | 3.1 | 0.09 | 0.03 | 3 | 0.06 | 0.02 | 3.19 | 0.29 | 0.09 |
| **Dmin** | 0.11 | 0 | 0.03 | 0.11 | 0 | 0 | 0.09 | 0 | 0.03 | 0.1 | 0 | 0.03 | 0.11 | 0 | 0.01 | 0.1 | 0.01 | 0.06 | 0.11 | 0 | 0.03 | 0.11 | 0.01 | 0.06 |
| **Dmax** | 0.8 | 0.01 | 0.02 | 0.96 | 0.05 | 0.06 | 0.84 | 0.03 | 0.04 | 0.87 | 0.03 | 0.04 | 0.8 | 0.07 | 0.08 | 0.81 | 0.06 | 0.07 | 0.83 | 0.03 | 0.04 | 0.74 | 0.06 | 0.09 |
| **IBD** | 2.6 | 0.11 | 0.04 | 2.71 | 0.33 | 0.12 | 2.53 | 0.31 | 0.12 | 1.72 | 0.11 | 0.06 | 2.8 | 0.51 | 0.18 | 2.31 | 1.15 | 0.5 | 3.15 | 0.95 | 0.3 | 2.64 | 0.13 | 0.05 |
| **Dldm** | 0.41 | 0 | 0.01 | 0.34 | 0.05 | 0.16 | 0.32 | 0.03 | 0.1 | 0.33 | 0.01 | 0.03 | 0.36 | 0.02 | 0.06 | 0.43 | 0.03 | 0.06 | 0.36 | 0.05 | 0.13 | 0.43 | 0.02 | 0.05 |
| **VarD** | 0.14 | 0.01 | 0.08 | 0.15 | 0.01 | 0.08 | 0.16 | 0.01 | 0.05 | 0.15 | 0 | 0 | 0.16 | 0.04 | 0.24 | 0.14 | 0 | 0.02 | 0.13 | 0.02 | 0.13 | 0.13 | 0.01 | 0.07 |
| **ELT** | 1.28 | 0.3 | 0.23 | 1.22 | 0.29 | 0.24 | 1.36 | 0.22 | 0.16 | 1.37 | 0.05 | 0.04 | 1.37 | 0.09 | 0.06 | 1.28 | 0.02 | 0.01 | 1.52 | 0.31 | 0.2 | 1.63 | 0.09 | 0.06 |
