# Supplementary material for: Genotypic diversity and plasticity of root system architecture to nitrogen availability in oilseed rape
Source: PLoS One. 2021 May 20;16(5):e0250966. doi: 10.1371/journal.pone.0250966 (PMC8136655; doi:10.1371/journal.pone.0250966)
Supplement: S4 Table — Mean, standard deviation (sd) and variation coefficient (cv) were calculated per genotype for each trait (n = 2). (DOCX) [file pone.0250966.s007.docx]

S4 Table: Descriptive statistics of N- plants. Mean, standard deviation (sd) and variation coefficient (cv) were calculated per genotype for each trait (n=2).

| Genotype | **AMBER** | | | **AVISO** | | | **CRESOR** | | | **EMIL** | | | **GASPARD** | | | **MILENA** | | | **MOHICAN** | | | **TOSCA** | | |
| --- | --- | --- | --- | --- | --- | --- | --- | --- | --- | --- | --- | --- | --- | --- | --- | --- | --- | --- | --- | --- | --- | --- | --- | --- |
| **Trait** | *mean* | *sd* | *cv* | *mean* | *sd* | *cv* | *mean* | *sd* | *cv* | *mean* | *sd* | *cv* | *mean* | *sd* | *cv* | *mean* | *sd* | *cv* | *mean* | *sd* | *cv* | *mean* | *sd* | *cv* |
| **TDB** | 0.67 | 0.1 | 0.15 | 0.57 | 0.07 | 0.11 | 0.76 | 0.05 | 0.07 | 0.59 | 0 | 0 | 0.63 | 0.17 | 0.27 | 0.6 | 0.2 | 0.33 | 0.76 | 0.07 | 0.09 | 0.72 | 0.08 | 0.11 |
| **RDB** | 0.26 | 0.02 | 0.1 | 0.21 | 0 | 0.01 | 0.3 | 0.1 | 0.32 | 0.22 | 0 | 0.01 | 0.26 | 0.13 | 0.52 | 0.23 | 0.12 | 0.53 | 0.31 | 0.04 | 0.13 | 0.32 | 0.08 | 0.25 |
| **LA** | 83.35 | 0.16 | 0 | 81.36 | 1.27 | 0.02 | 76.19 | 14.24 | 0.19 | 88.93 | 10.88 | 0.12 | 94.24 | 4.23 | 0.04 | 83.19 | 10.7 | 0.13 | 92.99 | 5.35 | 0.06 | 103.4 | 14.45 | 0.14 |
| **RS** | 0.72 | 0.19 | 0.26 | 0.56 | 0.09 | 0.16 | 0.63 | 0.22 | 0.34 | 0.65 | 0.08 | 0.12 | 0.85 | 0.54 | 0.63 | 0.59 | 0.17 | 0.29 | 0.79 | 0.26 | 0.33 | 0.78 | 0.08 | 0.1 |
| **RTD** | 0.11 | 0.03 | 0.28 | 0.11 | 0.01 | 0.05 | 0.13 | 0.05 | 0.37 | 0.14 | 0.04 | 0.26 | 0.15 | 0.05 | 0.36 | 0.11 | 0 | 0.03 | 0.11 | 0.02 | 0.19 | 0.14 | 0 | 0.03 |
| **NUtE** | 52.03 | 2.98 | 0.06 | 48.05 | 6.65 | 0.14 | 58.25 | 8.3 | 0.14 | 52.7 | 0.15 | 0 | 52.45 | 9.64 | 0.18 | 50.74 | 15.83 | 0.31 | 61.47 | 5.09 | 0.08 | 56.42 | 7.38 | 0.13 |
| **NUpE** | 0.41 | 0.07 | 0.17 | 0.42 | 0 | 0 | 0.46 | 0.04 | 0.09 | 0.36 | 0.06 | 0.17 | 0.42 | 0.02 | 0.04 | 0.42 | 0 | 0 | 0.41 | 0 | 0 | 0.45 | 0.02 | 0.05 |
| **CC** | 27.78 | 1.71 | 0.06 | 30.07 | 0.88 | 0.03 | 26.75 | 3.39 | 0.13 | 26.07 | 0.36 | 0.01 | 24.67 | 5.75 | 0.23 | 28.14 | 2.97 | 0.11 | 24.04 | 0.54 | 0.02 | 25.95 | 2.73 | 0.11 |
| **NC** | 2.12 | 0.21 | 0.1 | 2.11 | 0.27 | 0.13 | 1.91 | 0.04 | 0.02 | 2.07 | 0.04 | 0.02 | 1.9 | 0.41 | 0.22 | 2.2 | 0.4 | 0.18 | 1.75 | 0.52 | 0.29 | 1.97 | 0.07 | 0.03 |
| **Dmin** | 0.11 | 0 | 0.01 | 0.11 | 0 | 0.01 | 0.1 | 0 | 0.04 | 0.1 | 0 | 0.03 | 0.1 | 0 | 0.02 | 0.1 | 0.01 | 0.07 | 0.11 | 0 | 0.01 | 0.11 | 0.01 | 0.08 |
| **Dmax** | 0.76 | 0.17 | 0.23 | 0.8 | 0.01 | 0.01 | 0.65 | 0.03 | 0.05 | 0.65 | 0.01 | 0.01 | 0.69 | 0.04 | 0.06 | 0.64 | 0.05 | 0.08 | 0.67 | 0.03 | 0.04 | 0.69 | 0.05 | 0.07 |
| **IBD** | 4.54 | 0.81 | 0.18 | 3.83 | 0.53 | 0.14 | 5.2 | 0.27 | 0.05 | 3.27 | 0.95 | 0.29 | 4 | 0.69 | 0.17 | 5.05 | 0.34 | 0.07 | 5.32 | 0.01 | 0 | 3.17 | 0.6 | 0.19 |
| **Dldm** | 0.33 | 0.01 | 0.03 | 0.34 | 0.02 | 0.07 | 0.28 | 0.02 | 0.08 | 0.32 | 0.03 | 0.08 | 0.31 | 0.03 | 0.11 | 0.33 | 0.03 | 0.09 | 0.3 | 0.03 | 0.1 | 0.39 | 0.01 | 0.03 |
| **VarD** | 0.17 | 0.01 | 0.08 | 0.15 | 0.01 | 0.05 | 0.2 | 0 | 0.02 | 0.17 | 0.02 | 0.14 | 0.16 | 0.01 | 0.06 | 0.22 | 0.04 | 0.19 | 0.15 | 0 | 0.02 | 0.15 | 0 | 0 |
| **ELT** | 1.5 | 0.76 | 0.51 | 1.17 | 0.13 | 0.11 | 1.4 | 0.17 | 0.12 | 1.56 | 0.67 | 0.43 | 1.14 | 0.17 | 0.15 | 1.55 | 0.53 | 0.34 | 1.45 | 0.2 | 0.14 | 1.43 | 0.08 | 0.06 |
