# Supplementary material for: Genotypic diversity and plasticity of root system architecture to nitrogen availability in oilseed rape
Source: PLoS One. 2021 May 20;16(5):e0250966. doi: 10.1371/journal.pone.0250966 (PMC8136655; doi:10.1371/journal.pone.0250966)
Supplement: S6 Table — Pearson correlation coefficients are indicated in each cell, as well as the level of significance of the two by two correlations (***, Pvalue < 0.001; **, Pvalue < 0.01; *, Pvalue < 0.05; ns, non significant). (DOCX) [file pone.0250966.s009.docx]

*S6 Table: Correlations analysis between all the 15 measured traits in N- plants. Pearson correlation coefficients are indicated in each cell, as well as the level of significance of the two by two correlations (***, Pvalue < 0.001; **, Pvalue < 0.01; *, Pvalue < 0.05; ns, non significant).*

|  | **TDB** | **RDB** | **LA** | **RS** | **RTD** | **NUtE** | **NUpE** | **CC** | **NC** | **Dmin** | **Dmax** | **IBD** | **Dldm** | **VarD** | **ELT** |
| --- | --- | --- | --- | --- | --- | --- | --- | --- | --- | --- | --- | --- | --- | --- | --- |
| **TDB** | 1 (***) | 0.91 (***) | 0.02 (ns) | 0.55 (*) | 0.26 (ns) | 0.93 (***) | 0.38 (ns) | -0.63 (**) | -0.81 (***) | 0.22 (ns) | -0.03 (ns) | 0.32 (ns) | -0.14 (ns) | 0.2 (ns) | -0.23 (ns) |
| **RDB** | 0.91 (***) | 1 (***) | 0.08 (ns) | 0.74 (**) | 0.49 (ns) | 0.91 (***) | 0.21 (ns) | -0.79 (***) | -0.72 (**) | 0.27 (ns) | -0.11 (ns) | 0.16 (ns) | -0.02 (ns) | 0.17 (ns) | -0.18 (ns) |
| **LA** | 0.02 (ns) | 0.08 (ns) | 1 (***) | 0.17 (ns) | 0 (ns) | 0 (ns) | 0.13 (ns) | -0.28 (ns) | -0.04 (ns) | 0.21 (ns) | -0.2 (ns) | -0.46 (ns) | 0.32 (ns) | -0.57 (*) | 0.31 (ns) |
| **RS** | 0.55 (*) | 0.74 (**) | 0.17 (ns) | 1 (***) | 0.77 (***) | 0.59 (*) | -0.14 (ns) | -0.85 (***) | -0.64 (**) | 0.16 (ns) | -0.24 (ns) | 0.13 (ns) | -0.17 (ns) | -0.1 (ns) | -0.04 (ns) |
| **RTD** | 0.26 (ns) | 0.49 (ns) | 0 (ns) | 0.77 (***) | 1 (***) | 0.38 (ns) | -0.33 (ns) | -0.67 (**) | -0.41 (ns) | -0.22 (ns) | -0.33 (ns) | -0.02 (ns) | -0.15 (ns) | -0.05 (ns) | -0.09 (ns) |
| **NUtE** | 0.93 (***) | 0.91 (***) | 0 (ns) | 0.59 (*) | 0.38 (ns) | 1 (***) | 0.1 (ns) | -0.75 (***) | -0.82 (***) | 0.31 (ns) | -0.15 (ns) | 0.26 (ns) | -0.1 (ns) | 0.19 (ns) | -0.19 (ns) |
| **NUpE** | 0.38 (ns) | 0.21 (ns) | 0.13 (ns) | -0.14 (ns) | -0.33 (ns) | 0.1 (ns) | 1 (***) | 0.17 (ns) | -0.2 (ns) | -0.16 (ns) | 0.25 (ns) | 0.03 (ns) | 0.11 (ns) | 0.1 (ns) | -0.11 (ns) |
| **CC** | -0.63 (**) | -0.79 (***) | -0.28 (ns) | -0.85 (***) | -0.67 (**) | -0.75 (***) | 0.17 (ns) | 1 (***) | 0.62 (*) | -0.14 (ns) | 0.41 (ns) | -0.12 (ns) | 0.23 (ns) | 0.05 (ns) | -0.05 (ns) |
| **NC** | -0.81 (***) | -0.72 (**) | -0.04 (ns) | -0.64 (**) | -0.41 (ns) | -0.82 (***) | -0.2 (ns) | 0.62 (*) | 1 (***) | -0.21 (ns) | 0.04 (ns) | -0.23 (ns) | 0.26 (ns) | 0.03 (ns) | 0.36 (ns) |
| **Dmin** | 0.22 (ns) | 0.27 (ns) | 0.21 (ns) | 0.16 (ns) | -0.22 (ns) | 0.31 (ns) | -0.16 (ns) | -0.14 (ns) | -0.21 (ns) | 1 (***) | 0.44 (ns) | -0.21 (ns) | 0.43 (ns) | -0.39 (ns) | -0.16 (ns) |
| **Dmax** | -0.03 (ns) | -0.11 (ns) | -0.2 (ns) | -0.24 (ns) | -0.33 (ns) | -0.15 (ns) | 0.25 (ns) | 0.41 (ns) | 0.04 (ns) | 0.44 (ns) | 1 (***) | -0.32 (ns) | 0.42 (ns) | -0.22 (ns) | -0.62 (*) |
| **IBD** | 0.32 (ns) | 0.16 (ns) | -0.46 (ns) | 0.13 (ns) | -0.02 (ns) | 0.26 (ns) | 0.03 (ns) | -0.12 (ns) | -0.23 (ns) | -0.21 (ns) | -0.32 (ns) | 1 (***) | -0.6 (*) | 0.5 (*) | 0.07 (ns) |
| **Dldm** | -0.14 (ns) | -0.02 (ns) | 0.32 (ns) | -0.17 (ns) | -0.15 (ns) | -0.1 (ns) | 0.11 (ns) | 0.23 (ns) | 0.26 (ns) | 0.43 (ns) | 0.42 (ns) | -0.6 (*) | 1 (***) | -0.27 (ns) | -0.04 (ns) |
| **VarD** | 0.2 (ns) | 0.17 (ns) | -0.57 (*) | -0.1 (ns) | -0.05 (ns) | 0.19 (ns) | 0.1 (ns) | 0.05 (ns) | 0.03 (ns) | -0.39 (ns) | -0.22 (ns) | 0.5 (*) | -0.27 (ns) | 1 (***) | -0.13 (ns) |
| **ELT** | -0.23 (ns) | -0.18 (ns) | 0.31 (ns) | -0.04 (ns) | -0.09 (ns) | -0.19 (ns) | -0.11 (ns) | -0.05 (ns) | 0.36 (ns) | -0.16 (ns) | -0.62 (*) | 0.07 (ns) | -0.04 (ns) | -0.13 (ns) | 1 (***) |
